# Emotional, Behavioural and Executive Functioning Problems in Children in Residential Care

**DOI:** 10.3390/ijerph17103596

**Published:** 2020-05-20

**Authors:** Juan Manuel Moreno-Manso, María Elena García-Baamonde, Eloísa Guerrero-Barona, María José Godoy-Merino, Natalia Bueso-Izquierdo, Mónica Guerrero-Molina

**Affiliations:** 1 Department of Psychology, University of Extremadura, 06006 Badajoz, Spain; mgarsan@unex.es (M.E.G.-B.); eloisa@unex.es (E.G.-B.); nbueso@unex.es (N.B.-I.); monicagm@unex.es (M.G.-M.); 2 Department of Educational Sciences, University of Extremadura, 06006 Badajoz, Spain; godoymerino21@unex.es

**Keywords:** executive functions, behavioural and emotional problems, neuropsychology, psychological disorders, residential care

## Abstract

This research analyses the emotional and behavioural problems, as well as the problems in the executive functions, of children in residential care under protective measures, between 8 and 12 years of age. We analyse the relationship between the problems with their executive functions and their emotional and behavioural problems, as well as the predictive value of the executive functions for the said emotional and behavioural problems. The instruments used were as follows: five digits test (FDT), behavioural assessment of the dysexecutive syndrome in children (BADS-C) and the system of evaluation for children and adolescents (SENA). The results indicate that the children have difficulties in their executive functions, with such problems as in attention control and regulation, impulsiveness, mental rigidity, behavioural organisation and planning and resolving problems. They also have internalising and externalising problems, as well as difficulties in controlling their emotional reactions and understanding the emotions of others. It becomes evident that the difficulties in their executive functions are related to and predict their emotional and behavioural problems. The research demonstrates the need to intervene in the problems detected through the design of therapeutic programmes and interventions in the residential context.

## 1. Introduction

Children in the child protection scheme are especially vulnerable to cognitive, emotional and behavioural problems. The abuse and neglect that these children have experienced, together with the fact that many of them come from conflictive family environments or environments where affection may be lacking, are risk factors for the apparition of different types of disorder [1,2,3].

Among the cognitive and emotional repercussions of the children suffering neglect, what stands out are the difficulties in the capacity for processing social information, the impulsive cognitive style, the scarce capacity for resolving problems by analysing the situation and looking for alternatives, difficulties in attention control, in regulating one’s emotions and in taking decisions, the lack of planning behaviour and anticipating consequences, the scarce tolerance of frustration, the lack of cognitive flexibility and learning difficulties [4,5,6,7].

Similarly, there are several works that demonstrate the greater risk of suffering psychopathologies and behavioural problems in children who experience abuse and neglect [8,9,10]. Among the disorders, we can find behavioural patterns both externalising (oppositionist behaviour, aggressiveness, criminal behaviour, hyperactivity, etc.) and internalising (anxiety, depression, post-traumatic stress disorder, etc.). Some studies point to a greater prevalence of externalising behaviour [11]. Nevertheless, other research works demonstrate a high incidence of internalising behaviour [12], or even both in equal proportions [13,14].

The adverse life experiences that these children go through can affect the neurodevelopment of their brains [15,16,17]. Works in neurobiological research have shown the structural alterations that can occur within the brain (hippocampus, amygdala, cerebellum, corpus callosum and cerebral cortex) when a person is exposed to stressful situations with a very high intensity, such as abuse or parental violence [18,19].

Davis, Moss, Nogin & Webb [20] point out that the cognitive, emotional and behavioural consequences for children who are exposed to situations of abuse during childhood can be explained by dysfunctions in the prefrontal regions, altering the neuropsychological functioning and inhibiting neurogenesis. It is in the prefrontal regions that we acquire cognitive flexibility [21,22], control of our impulses and the ability to regulate our emotions [5,23], behaviour planning and decision-making [20], as well as the capacity to direct our own behaviour through learning from past experience [24].

The structural changes in the brain are related to the greater vulnerability of children to certain psychopathologies, as well as having difficulties in learning, attention, memory, language, intelligence, mental flexibility, conflict resolution, motor control and regulating the emotions and controlling one’s impulses, as it is the executive functions which are responsible for supervising and coordinating the cognitive, emotional and behavioural processes that are essential for both children and adolescents [5,20,25,26,27].

In this context, the objectives of this study were: to analyse the emotional and behavioural problems, as well as the problems in the executive functions shown by a sample of Spanish children in residential care with protective measures; to examine the relation between the problems in the executive functions and the children’s behavioural and emotional problems; and also to determine the predictive value of the executive functioning for the emotional and behavioural problems. On the basis of the theoretical review carried out, we expected the children to show behavioural and emotional problems as well as problems in their executive functions (hypothesis 1). In addition, we also expected that the difficulties present in the executive functions would be related to their behavioural and emotional problems (hypothesis 2). We also anticipated that the problems in the executive functions would act as predictors for the subjects’ emotional and behavioural difficulties (hypothesis 3).

## 2. Materials and Methods

### 2.1. Participants

The emotional and behavioural problems, as well as problems in the executive functions, were analysed for a total of 37 children with protective measures in residential care. There were 23 males (66.7%) and 14 females (33.3%) between 8 and 12 years of age. The participants were the total number of children in that age range in the residential care centres of the region of Extremadura (Spain) in 2019, including private residential care centres. The average length of the children’s institutionalisation was 27 months. The sample is small, as children between 7 and 14 years of age in Spain only make up 31% of residential care cases. The predominant age group is that of 15–17 years of age (63%) [28]. Residential care in Spain is a temporary protective measure whose final goal is a return to the family of origin or to a new family.

The participants were children who were in residential care due to the parents’ neglect or renunciation; children who had suffered physical and/or psychological abuse, sexual abuse and serious physical neglect; also children who had been separated from their family because the parents/carers could not carry out their parental duties (serious mental disorder, drug consumption and/or prostitution).

When children enter residential care, the regional social services carry out an evaluation of their situation of lack of protection, as well as of the type of abuse. It is during this evaluation that emotional and cognitive difficulties are observed in some of the children as a consequence of their social and family environment and of the abuses suffered. Thus, the need for an evaluation of the children’s mental health was proposed in order to detect the presence of cognitive, emotional and behavioural problems, as well as to evaluate the need for therapeutic assistance.

Nevertheless, González-García et al. [9] point out a fact that should be considered: as the stay in residential care is prolonged, the emotional and/or behavioural disruption could become greater.

The children of immigrants were excluded from the research as this would have supposed an important bias due to a lack of knowledge of the language. Further excluded were children diagnosed with an intellectual disability, with any kind of autism and those with attention deficit and hyperactivity. Finally, we also excluded any children who were subject to emergency measures or were in the process of evaluation.

### 2.2. Instruments

#### 2.2.1. Five Digits Test

The five digits test (FDT) [29] was used to evaluate the speed of cognitive processing and specific aspects of attention and the executive functions, such as attention control, cognitive flexibility, efficiency in alternation between mental processes, the capacity for inhibition and adaptation to new situations. The test consists of 4 tasks of progressive difficulty: reading digits, counting asterisks, choosing and alternating. As for the normative data of this instrument, the average scores were between 40 and 70. The reliability reflects adequate indices, since Cronbach’s Alpha for all the sub-scales are within the range α = 0.80 and α = 0.94.

#### 2.2.2. Behavioural Assessment of the Dysexecutive Syndrome in Children (BADS-C)

Based on the objectives of our study, we applied two subtasks from BADS-C [30]: Zoo Map and Playing Cards. The Zoo Map test evaluates the ability to plan, organise and resolve problems to achieve a goal. The Playing Cards test measures the subject’s capacity to respond correctly to a rule and to change her/his response depending on the rule in question. The test evaluates tendencies in perseverance or mental flexibility, speed of processing, the capacity to adapt to new situations and the ability to follow externally imposed strategies. This instrument has been designed to evaluate the executive functions starting from the following statistical cut-off points: a normal average score is considered to be 3–4; while a score equal to or lower than 2 is indicative of some degree of deterioration. The reliability reflects adequate indices, since Cronbach’s Alpha for Zoo Map is α = 0.96 and for Playing Cards is α = 0.91.

#### 2.2.3. System of Evaluation for Children and Adolescents (SENA)

This is an instrument aimed at detecting emotional and behavioural problems in children and adolescents [31]. It evaluates the presence of interiorised and exteriorised problems, contextual problems (family, school, companions), areas of vulnerability that may contribute to the start or continuation of some problems, and the children’s psychological resources to face difficulties. As for the normative data of this instrument, the average is 50 and the standard deviation 10. The reliability reflects adequate indices, as the average Cronbach’s Alpha is between α = 0.82 and α = 0.85 on the scales in samples from the general and clinical population, respectively, and of α = 0.93 in the global indices.

### 2.3. Procedure

The research was authorised and approved by the institution in charge of the children’s tutorship (Region of Extremadura, Spain), as their legal representative. All subjects gave their informed consent for inclusion before they participated in the study. All procedures performed were in accordance with the ethical standards of Extremadura University (Ref. 103/2018) and with the 1964 Helsinki declaration and its later amendments or comparable ethical standards.

The instruments were applied, in an individual format, by two evaluators in the residential care centres. The children participated on a voluntary basis. The evaluators were given instructions prior to the application of the instruments in order to guarantee the validity and reliability of the data gathering. No difficulties of understanding or manipulation of the instruments during their application were reported.

### 2.4. Data Analysis

First, we carried out a descriptive analysis of the behavioural and emotional problems, as well as of the problems in the children’s executive functions. Having determined that it was appropriate to use the non-parametric tests, we then carried out Spearman’s correlational analysis to find the relation between the difficulties in the executive functions and the emotional and behavioural problems; finally, we performed a linear regression analysis to determine the extent to which the executive functions can predict the children’s emotional and behavioural problems.

The statistical package SPSS version 25 was used for the statistical treatment of the data.

## 3. Results

With regard to the executive functions, Table 1 shows the descriptive data from the FDT and the BADS-C.

The results of the FDT indicate that the children score below average in reading, counting, choice and alternation. Thus, they have difficulties in the automatic cognitive processes (reading and counting) and in those processes that require active mental control (choice and alternation). In response inhibition and mental flexibility, the scores are also below average. The children have difficulties in inhibiting behaviour patterns and adapting to new situations, in processing information, in mental flexibility and in attention control and regulation.

As for the BADS-C, the results indicate that the average scores are below what is considered normal in the zoo map and in playing cards. We find difficulties in the tasks relating to organisation, planning, problem solving and decision taking.

With respect to the emotional and behavioural problems, Table 2 shows the descriptive data from the SENA.

With respect to the control scales, the results are within the normal range in inconsistency, negative impression and positive impression.

As for the global indices, they obtained higher than average scores in all the indices (global, emotional, behavioural, executive functions and contextual). The highest score was in the index of problems in the executive functions, which seems to be one of the most outstanding difficulties. In the index of personal resources, the score is within the normal range, although the average is rather low.

With respect to the problem Scales, the results indicate higher than average scores in such interiorised problems as anxiety, social anxiety and somatic problems. As for the scales of exteriorised problems, the highest scores are in attention problems and hyperactivity-impulsiveness. There are also higher than average scores in defiant behaviour and rage control problems. In contextual problems, difficulties are seen in school and companions, indicating that these are the most complicated contexts for the children.

In the Vulnerability scale, they have problems controlling emotions, and this could be a risk factor.

As for the Personal Resources scales, the levels of self-esteem and integration & social competence are normal, but the average scores are rather low, becoming problematic in several children.

As for the relationship between the difficulties in the executive functions and the emotional and behavioural problems, Table 3 shows the correlation data.

The results show a significant correlation between all the global indices and the results for the executive functioning. Thus, the presence of emotional and behavioural problems, as well as in the executive functions, correlates with the difficulties in choice, alternation, response inhibition, mental flexibility and playing cards.

With respect to the interiorised problems, it is worth mentioning the correlation between anxiety and alternation, mental flexibility and playing cards. Similarly, we can also state that Post-traumatic symptomatology correlates with playing cards.

In the exteriorised problems, the results indicate significant correlations between attention problems, hyperactivity-impulsiveness and counting, choice, alternation, response inhibition, mental flexibility, zoo map and playing cards. Defiant behaviour also correlates with choice, alternation, response inhibition, mental flexibility and playing cards. There is also a correlation between aggression and zoo map.

In contextual problems, the data indicate a significant correlation between problems with companions and alternation.

In the vulnerability scale, there is a significant correlation between problems controlling emotions and counting, choice, alternation, response inhibition, zoo map and playing cards.

While in the Personal resources scales, the results indicate a significant correlation between self-esteem and playing cards.

Finally, in order to identify whether the executive functions act as predictors of the children’s emotional and behavioural problems, we carried out a regression analysis (Table 4).

The results point to the cognitive processes involved in choice (β = 0.40; p = 0.034), alternation (β = 0.56; p = 0.002), response inhibition (β = 0.41; p = 0.031), mental flexibility (β = 0.48; p = 0.011) and playing cards (β = −0.56; p = 0.002) as predictors of the global Index of problems.

As for the index of emotional problems, the following processes appear as predictors: counting (β = 0.54; p = 0.003), choice (β = 0.52; p = 0.005), alternation (β = 0.54; p = 0.004), response inhibition (β = 0.44; p = 0.021), zoo map (β = −0.49; p = 0.009) and playing cards (β = −0.54; p = 0.004).

With respect to the index of behavioural problems, the mental processes involved in counting (β = 0.45; p = 0.016), choice (β = 0.55; p = 0.003), alternation (β = 0.54; p = 0.003), response inhibition (β = 0.49; p = 0.008), mental flexibility (β = 0.40; p = 0.038), zoo map (β = −0.48; p = 0.011) and playing cards (β = −0.50; p = 0.007) are outstanding as predictors.

Finally, in the index of problems in the executive functions, the processes involved in counting (β = 0.39; p = 0.040), choice (β = 0.44; p = 0.021), alternation (β = 0.58; p = 0.002), response inhibition (β = 0.40; p = 0.039) and mental flexibility (β = 0.48; p = 0.010) turn out to be predictors.

## 4. Discussion

On the basis of the results of the study, we can conclude that the children under protective measures have emotional and behavioural problems, as well as difficulties with their executive functions.

The children in residential care have difficulties related to various components of their executive functions, such as problems with attention control and regulation (they are easily distracted and find it difficult to concentrate); problems in controlling their behaviour and inhibiting inappropriate or ineffective behaviour patterns (impulsiveness); difficulties adapting to new situations, different contexts or demands made to them (scarce mental flexibility and problems inhibiting automatic responses); also, deficits in information processing, they process information more slowly and have to make an effort to focus their attention, they tire easily.

As for resolving problems, the children have difficulties in finding alternatives, showing a scarce ability to face situations that involve cognitive stress. Their scare capacity for organisation, planning and problem solving can be a handicap in the activities of daily life in the residential care centre. The children have problems in initiating a task, supervising its execution and using this information to adjust their behaviour to what is asked of them, as well as a scarce vision of the activity as a whole.

The scarce capacity for planning behaviour may make the decision-making process more difficult, as well as their social competence [6,20,32]. As observed in our study, the children possess scarce personal resources to face situations and difficulties to integrate into social groups and to comply with the norms. Their social competence and degree of personal satisfaction are not appropriate, becoming a hindrance to their social interaction and friendship relationships.

Several studies [2,22,33,34] with victims of child abuse also brought to light problems in their neurocognitive functioning, affecting their capacity for cognitive flexibility and problem solving.

As for behavioural problems, our study concludes that the children have an above average level of affectation and unease. The children’s behaviour is unadapted, which can be seen in both internalising and externalising problems.

With regards to the internalising problems, worth mentioning are the problems of anxiety. The children have persistent worries, overreactions and are highly nervous. The higher levels of anxiety in children in residential care has also been shown to be true in several studies [12,13,35].

With regards to the externalising problems, we found the presence of symptoms showing a lack of attention and hyperactive and impulsive behaviour, characteristic of the attention deficit hyperactivity disorder (ADHD). The children show a high level of motor activation and difficulties in inhibiting, controlling and regulating their behaviour. Defiant behaviour, characterised by opposition to figures of authority and disobedience, was also observed in several of the children. In this sense, the children express a negative attitude and dissatisfaction with school, also perceiving a lack of support and tension in the relationships with some of their companions. Several children expressed unease concerning the residential care centre and the educators. As with our study, various works of research [11,36] have also demonstrated a greater presence of externalising as opposed to internalising behaviour patterns. Others [21,37] have found concentration problems in victims of child abuse, thus causing a decrease in the execution of proposed tasks and/or activities.

As for emotional problems, the children have difficulties in understanding and regulating their own emotions, as well as in understanding the emotions of others, at times showing difficulties in controlling their emotional reactions. Several studies [5,7,38,39,40] have also found difficulties in regulating emotions, in attuning emotionally with others and understanding the emotional states of others. They also found a lack of self-control and increased impulsiveness in victims of child abuse.

One important aspect to highlight from our study is that the executive functions are related to and predict the emotional and behavioural problems of the children in residential care. During the later stages of childhood and in adolescence, the areas of the brain that are developed are mainly those responsible for establishing social relationships and for resolving problems. Neurobiological studies show that during this period there is a sharp increase in the activity of the frontal regions and that, thanks to the development of the executive functions, the children are able to anticipate the consequences of their behaviour and that of others, thereby increasing their emotional control and learning to self-regulate. Then, at the start of adolescence, the execution of problem-solving tasks improves, as does the speed of processing information, the use of strategies and inhibitory control [18,41,42].

This study is not exempt from limitations. The research data come from a very specific context (residential care centres) and have a transversal nature. A longitudinal study would provide more evidence concerning emotional and behavioural problems, as well as the executive functions.

Similarly, in this research, it has only been possible to use self-reporting through the evaluation system of children and adolescents (SENA). For future work, the complete evaluation available in this system would be needed, integrating the information from informants (educators and teaching staff) of the children’s different development contexts.

In this study, it has not been possible to consider some variables that could modulate the evolution of the problems, such as the length of stay in residential care. In future research, it would be relevant to evaluate the presence of cognitive, emotional and behavioural problems since the children entered residential care. This would allow us to identify the intervening factors with greater accuracy, as well as being able to set up an early therapeutic response to the problems observed in the children.

## 5. Conclusions

Our study has permitted us to detect a wide spectrum of problems and areas of vulnerability in children under protective measures, which should be given priority attention. Several clinical and educational implications can be derived from the results obtained in this study.

It is essential to give therapeutic cover to the internalising and externalising problems of children in residential care. The study has shown a greater presence of externalising problems, such as concentration problems and hyperactivity-impulsiveness. Along the same lines, the difficulties found in the executive functions and the problems with emotion regulation are especially relevant.

In this sense, it would be particularly relevant to set up intervention programmes aimed at mitigating and modifying those aspects which could be most problematic in the residential context. The educational and therapeutic work carried out in the residential care centres is fundamental if we hope to achieve a better psychological adjustment and a better quality of life for these children. The care centre is the reference context for those children who are in a situation of neglect, and it should be precisely in the care centre where the tools and strategies should be set up for them to face life productively and allow them to grow and develop to their best possible potential. To do so, the collaboration of the professional staff of the centres is essential.

The intervention should be aimed at working with the cognitive, behavioural and emotional resources of the children, so that they can improve their capacity to resolve problems. Training in real situations is essential, as this would allow them to find alternatives to solve the problems of daily life [43,44,45].

Training in self-instruction for the children, through the use of internal language, can promote the use of cognitive schemes to facilitate adaptive behaviour patterns. Thus, the children would be able to self-regulate their behaviour in their daily activities and their interactions with others.

Training in solving the problems of everyday life will also have an effect on their executive functioning, giving them a greater capacity for reflection and analysis before acting, a greater tolerance in interpersonal relations, less impulsiveness and a greater capacity for planning their behaviour and understanding the consequences of their actions.

Similarly, we must also highlight the need for the emotional training of these children, in order to provide them with self-knowledge and control of their emotions, as well as the recognition and understanding of the emotions of others. The intelligent use of the emotions is the key to an adaptive style of facing up to life and to a child’s mental health; thus, the importance of working on their interpersonal and intrapersonal emotional competence in the residential context.

We trust that the present research can serve to encourage future studies in greater depth.

## Figures and Tables

**Table 1:** Means and standard deviations of the five digit test (FDT) and behavioural assessment of the dysexecutive syndrome in children (BADS-C).

	M	SD	Min, Max	IC 95%		Cronbach’s Alpha
FDT						
Reading	17.52	18.54	1, 50	10.19	24.85	0.80
Counting	13.96	12.61	1, 45	8.97	18.95	0.82
Choice	17.37	21.79	1, 65	8.75	25.99	0.85
Alternation	17.11	22.45	1, 85	8.23	25.99	0.86
Response inhibition	27.59	28.27	1, 90	16.41	38.78	0.85
Mental flexibility	24.96	30.00	1, 85	13.10	36.83	0.86
BADS-C						
Zoo Map	1.89	0.32	1, 2	1.76	2.02	0.90
Playing Cards	1.41	0.50	1, 2	1.21	1.61	0.87

**Table 2:** Means and standard deviations of the system of evaluation for children and adolescents (SENA).

	M	SD	Min, Max	IC 95%		Cronbach’s Alpha
Control Scales						
Inconsistency	51.70	6.36	36, 70	49.19	54.22	-
Negative impression	53.78	7.59	32, 70	50.77	56.78	-
Positive impression	49.85	6.44	34, 58	47.30	52.40	-
Global Indices						
Global index of problems	62.85	4.11	55, 75	61.23	64.48	0.92
Index of emotional problems	62.00	6.11	53, 81	59.58	64.42	0.90
Index of behavioural problems	61.26	6.78	51, 81	58.57	63.94	0.90
Index of problems in the executive functions	68.52	4.45	62, 78	66.75	70.28	0.93
Index of contextual problems	63.48	5.36	54, 70	61.36	65.60	0.85
Index of personal resources	41.04	4.62	32, 52	39.21	42.86	0.80
Problem Scales						
*Interiorised problems*						
Depression	51.63	4.50	40, 60	49.85	53.41	0.81
Anxiety	64.07	7.39	54, 86	61.15	67.00	0.83
Social anxiety	61.96	5.00	53, 73	59.98	63.94	0.80
Somatic problems	61.85	6.68	43, 78	59.21	64.50	0.76
Post-traumatic symptomatology	56.81	7.10	46, 72	54.00	59.63	0.78
*Exteriorised problems*						
Attention problems	68.81	6.08	60, 84	66.41	71.22	0.86
Hyperactivity-impulsiveness	68.37	5.63	60, 82	66.14	70.60	0.86
Rage control problems	60.81	11.55	32, 92	56.24	65.39	0.80
Aggression	58.15	3.13	50, 66	56.91	59.39	0.78
Defiant behaviour	62.15	6.82	52, 82	59.45	64.85	0.76
*Contextual problems*						
Family problems	57.22	9.23	40, 67	53.57	60.88	0.77
School problems	61.41	3.79	50, 72	59.91	62.91	0.79
Problems with companions	60.52	5.04	48, 72	58.52	62.51	0.80
Vulnerability scale						
Problems controlling emotions	67.26	7.12	50, 86	64.44	70.08	0.82
Personal Resources scales						
Self-esteem	43.85	4.60	36, 58	42.03	45.67	0.80
Integration & social competence	42.67	3.67	36, 50	41.21	44.12	0.78

**Table 3:** Correlation analysis between the children’s emotional and behavioural problems and their executive functions.

	Reading	Counting	Choice	Alternation	Response Inhibition	Mental Flexibility	Zoo Map	Playing Cards
	*ρ*	*ρ*	*ρ*	*ρ*	*ρ*	*ρ*	*ρ*	*ρ*
Global indices								
GLO	0.005	0.145	0.313	0.462 *	0.477 *	0.449 *	−0.063	−0.634 **
EMO	0.120	0.262	0.388 *	0.500 **	0.496 **	0.441 *	−0.226	−0.642 **
CON	0.118	0.151	0.436 *	0.494 **	0.578 **	0.466 *	−0.254	−0.595 **
EJE	0.225	0.527 **	0.531 **	0.670 **	0.521 **	0.586 **	−0.333	−0.480 *
CTX	0.319	0.338	0.242	−0.039	0.040	−0.248	−0.253	−0.078
REC	−0.330	−0.199	−0.021	−0.105	0.036	−0.131	0.145	−0.103
Interiorised problems								
DEP	−0.042	0.031	−0.075	−0.216	−0.193	−0.214	0.070	0.254
ANS	−0.013	0.139	0.262	0.447 *	0.276	0.398 *	−0.101	−0.410 *
ASC	−0.189	−0.208	0.023	0.248	0.118	0.278	0.247	−0.223
SOM	0.175	0.285	0.254	0.231	0.084	0.069	−0.162	−0.148
PST	−0.089	−0.050	0.102	0.170	0.213	0.066	−0.183	−0.518 **
Exteriorised problems								
ATE	0.197	0.434 *	0.398 *	0.558 **	0.406 *	0.455 *	−0.509 **	−0.582 **
HIP	0.308	0.567 **	0.560 **	0.639 **	0.537 **	0.544 **	−0.516 **	−0.487 **
IRA	0.046	−0.096	−0.240	−0.188	−0.266	−0.179	0.244	0.146
AGR	−0.112	−0.098	−0.128	−0.107	0.034	0.012	−0.391 *	0.065
DES	0.146	0.174	0.463 *	0.521 **	0.590 **	0.497 **	−0.260	−0.558 **
Contextual problems								
FAM	0.184	0.283	0.114	0.140	0.137	0.086	−0.126	−0.272
ESC	0.087	0.148	0.022	−0.039	−0.078	−0.066	−0.048	0.108
COM	−0.050	−0.183	−0.322	−0.411 *	−0.380	−0.375	0.294	−0.068
Vulnerability scale								
REG	0.278	0.491 **	0.450 *	0.454 *	0.508 **	0.344	−0.529 **	−0.413 *
Personal Resources scales								
AUT	0.197	0.158	0.008	−0.087	−0.179	−0.259	−0.174	0.430 *
SOC	0.040	0.024	0.041	0.244	0.093	0.276	−0.046	−0.367

* *p* < 0.05; ** *p* < 0.01. GLO: global index of problems; EMO: index of emotional problems; CON: index of behavioural problems; EJE: index of problems in the executive functions; CTX: index of contextual problems; REC: index of personal resources; DEP: depression; ANS: anxiety; ASC: social anxiety; SOM: somatic problems; PST: post-traumatic symptomatology; ATE: attention problems; HIP: hyperactivity-impulsiveness; IRA: rage control problems; AGR: aggression; DES: defiant behaviour; FAM: family problems; ESC: school problems; COM: problems with companions; REG: problems controlling emotions; AUT: self-esteem; SOC: integration & social competence.

**Table 4:** Regression analysis between the emotional and behavioural problems and the executive functions.

	GLO				EMO				CON				EJE			
	R²	β	*t*	*Sig.*	R²	β	*t*	*Sig.*	R²	β	*t*	*Sig.*	R²	β	*t*	*Sig.*
FDT																
Reading	0.00	0.05	0.25	0.799	0.09	0.30	1.62	0.117	0.11	0.34	1.80	0.083	0.02	0.16	0.81	0.424
Counting	0.05	0.23	1.19	0.242	0.29	0.54	3.24	0.003	0.21	0.45	2.58	0.016	0.15	0.39	2.16	0.040
Choice	0.16	0.40	2.24	0.034	0.27	0.52	3.10	0.005	0.30	0.55	3.31	0.003	0.19	0.44	2.45	0.021
Alternation	0.31	0.56	3.41	0.002	0.29	0.54	3.22	0.004	0.29	0.54	3.26	0.003	0.33	0.58	3.55	0.002
Response inhibition	0.17	0.41	2.28	0.031	0.19	0.44	2.47	0.021	0.24	0.49	2.87	0.008	0.16	0.40	2.18	0.039
Mental flexibility	0.23	0.48	2.73	0.011	0.13	0.36	1.95	0.062	0.16	0.40	2.19	0.038	0.23	0.48	2.80	0.010
BADS-C																
Zoo map	0.06	−0.24	−1.27	0.215	0.24	−0.49	−2.81	0.009	0.23	−0.48	−2.74	0.011	0.11	−0.33	−1.77	0.087
Playing cards	0.32	−0.56	−3.44	0.002	0.29	−0.54	−3.21	0.004	0.25	−0.50	−2.94	0.007	0.14	−0.37	−2.01	0.055

GLO: global index of problems; EMO: index of emotional problems; CON: index of behavioural problems; EJE: index of problems in the executive functions.
